# Effects of Six Natural Compounds and Their Derivatives on the Control of Coccidiosis in Chickens

**DOI:** 10.3390/microorganisms12030601

**Published:** 2024-03-17

**Authors:** Yue Hou, Bo Han, Zehua Lin, Qizheng Liu, Zhenhe Liu, Hongbin Si, Dandan Hu

**Affiliations:** 1 College of Animal Science and Technology, Guangxi University, Nanning 530004, China; 2034130143@st.gxu.edu.cn (Y.H.); 2034130145@st.gxu.edu.cn (B.H.); 2034130138@st.gxu.edu.cn (Z.L.); 2134110315@st.gxu.edu.cn (Q.L.); 2134130207@st.gxu.edu.cn (Z.L.); shb2009@gxu.edu.cn (H.S.); 2 Guangxi Zhuang Autonomous Region Engineering Research Center of Veterinary Biologics, Nanning 530004, China; 3 Guangxi Key Laboratory of Animal Breeding, Disease Control and Prevention, Nanning 530004, China

**Keywords:** natural products, *Eimeria*, anticoccidials, microbiota, silymarin

## Abstract

Chicken coccidiosis costs the poultry industry over GBP 10 billion per year. The main method of preventing and controlling coccidiosis in chickens continues to be the use of drugs. Unfortunately, the prevalence of drug resistance in the field reduces or even eliminates the effectiveness of drugs, and drug residues in the food supply chain can also can be harmful to humans. Therefore, safe and effective anticoccidial drugs are urgently needed. Natural products have many advantages such as being safe, effective and inexpensive and are a sustainable way to control coccidiosis. In this study, the anticoccidial effects of six natural compounds were tested by *Eimeria tenella* infection. Oocyst production, cecum lesion, body weight gain, feed conversion ratio, and intestinal microbiota were measured. The results showed that nerolidol had a moderate effect on maintaining both body weight gain and feed conversion ratio. Silymarin and dihydroartemisinin showed significant anticoccidial effects by reducing total oocyst output. Dihydroartemisinin also significantly reduced the cecum lesion caused by *Eimeria* infection, but this compound may be toxic to the host at such informed doses because it decreases growth and survival rates. In addition, both silymarin and dihydroartemisinin partly restored the microbiota after challenge. This indicates that silymarin, dihydroartemisinin, and nerolidol are effective in the control of chicken coccidiosis. Our data provide basic knowledge about the anticoccidial effects of such natural compounds/derivates.

## 1. Introduction

Coccidiosis is caused by infection of the apicomplexan parasite *Eimeria* species in host intestinal epithelial cells and can lead to typhlitis, with cecal core formation, hematochezia, poor food conversion ratios, and mortality in farm animals, especially in chickens [1,2]. According to a 2016 calculation model, chicken coccidiosis causes economic losses of more than GBP 10 billion annually to the world poultry industry [3]. Different strategies are currently used to control the disease, such as improving management and hygiene, using anticoccidial products, or applying various types of anticoccidial vaccines. However, prophylactic application of coccidiostats in feed or drinking water remains the most widely distributed method for controlling coccidiosis globally [4]. 

Of all the anticoccidial drugs, chemically synthesized drugs are by far the most widely used because of their cost-effectiveness and potent anticoccidial efficiency. They have made a significant contribution to the control of coccidiosis in the poultry industry over the past 50 years. However, in recent years, more drug resistance has been detected/reported in field strains, and drug residues in poultry products are an important factor that cannot be ignored [5]. Although the use of chemical drugs remains an integral part of worldwide coccidiosis control, pressure from legislation and consumers to reduce drug administration in the food chain is forcing us to develop safe, green, and durable approaches to coccidiosis control [6,7,8]. 

Plant natural products are rich in sources and readily available on earth, some of which have been shown to be an effective antiparasitic drug with the advantages of low side effects, low drug residues, and low price [9]. Currently, many natural products have been shown to have some therapeutic effect against coccidiosis in chickens, but there is still room for improvement. The extract from the bark of the pine tree (*Pinus radiata*), which is rich in condensed tannins, has been reported to inhibit the life cycle of coccidia, as evidenced by decreased sporulation of oocysts in *E. tenella*, *E. maxima*, and *E. acervuline* [10]. Extracts of *Dichroa febrifuga* Lour have also been shown to reduce bloody diarrhea, intestinal lesions, and oocyst excretion as well as to increase BWG, which is effective as an anticoccidial to protect chickens from coccidiosis infection [11]. More natural compounds have yet to be explored for their anticoccidial effects.

Silymarin has good effects in terms of anticancer activity, regulation of apoptosis and inflammatory response [12,13,14], and protection and repair of liver damage [15,16]. It also significantly attenuates macroscopic colonic damage as well as reduces colonic myeloperoxidase activity. The beneficial effect is accompanied by an improvement in the colonic oxidative status, which is altered in colonic inflammation, via the prevention of glutathione depletion and a reduction in malonyldialdehyde production [17]. Notably, silymarin is an antioxidant without side effects even at relatively high physiological dosage [18]. Artemisinin, as a frontline antimalarial drug, has been reported to have some anticoccidial activity [2,19,20]. Dihydroartemisinin, a derivate of artemisinin with stronger antimalarial activity than artemisinin [21], has been demonstrated to be an effective and fast-acting antimalarial drug with low toxicity in vitro and in vivo [22]. Quercetin and genistein could alleviate the gut inflammatory responses of chickens and modulate gut microbiota. Resveratrol sustains intestinal barrier integrity, improves antioxidant capacity, and alleviates inflammation [23]. Nerolidol attenuates oxidative stress and inflammation in acetic-acid-induced colitis [24]. Quercetin, resveratrol, genistein, and nerolidol all exert inhibitory effects against other apicomplexan protozoan parasites, such as *Toxoplasma gondii* or *Plasmodium falciparum* [25,26,27,28].

These plant natural compounds have shown potential inhibitory effects on antiprotozoan parasites, but their role in the control of coccidiosis caused by Eimeria infections in chickens is still unknown. As a result, six plant natural compounds were tested for their anticoccidial effects, including silymarin, dihydroartemisinin, genistein, resveratrol, quercetin, and nerolidol. The experimental results showed that silymarin, dihydroartemisinin, and nerolidol had moderate anticoccidial effects, providing new alternatives for the prevention and control of coccidiosis under certain concentrations and facilitating their translation into practical applications for poultry husbandry.

## 2. Materials and Methods

### 2.1. Ethical Statement

The use of animals in this study was approved by the Administration Committee of Laboratory Animals in Guangxi University and was performed in accordance with the Institutional Animal Care and Use Committee guidelines (approval number: Gxu-2021-013).

### 2.2. Animals

One-day-old Sanhuang chickens were purchased from Fufeng Animal Husbandry Co. LTD, Nanning, China, and raised in a coccidia-free environment. Filtered water and feed free of anticoccidials and antibiotics were provided ad libitum. During the experiment, chickens were reared in individual cages with dividers underneath to prevent contact with feces. To ensure that chickens were not infected with coccidia prior to inoculation, feces were monitored for one week using the fecal suspension method to ensure that they were free of coccidia oocysts.

### 2.3. Parasites

The *E. tenella* Houghton (ETH) strain was used to infect chickens. This strain was maintained and propagated by oral infection in chickens, and oocysts were sporulated in a 2.5% solution of potassium dichromate. The number of oocysts was determined using an advanced McMaster’s chamber, and the sporulation rate was observed and adjusted accordingly. Procedures for parasite collection, purification, and sporulation were previously described [29].

### 2.4. Compounds

Silymarin (min. 80% purity), dihydroartemisinin (min. 98% purity), nerolidol (min. 97% purity), quercetin (min. 95% purity), resveratrol (min. 99% purity), genistein (min. 97% purity), and diclazuril (min. 98% purity) were purchased from Aladdin (Shanghai, China) and introduced into chicken’s standard grower feed, respectively.

### 2.5. Experimental Design

One hundred and eight 5-day-old chickens were divided into nine groups of twelve birds each. Each bird was orally infected with 10,000 fresh sporulated *E. tenella* oocysts and treated with 75 ppm of dihydroartemisinin [30], 500 ppm of silymarin [31], 200 ppm of genistein [32], 1000 ppm of resveratrol [33], 1000 ppm of quercetin [34], and 500 ppm of nerolidol [35] in their feed or drinking water (nerolidol). The concentrations used for the tested compounds were selected with reference to previous reports on their use as feed additives or on their processes (maximal price of ~USD 500/ton). An infected and 1 ppm diclazuril-treated group was used as the positive control, infected and untreated birds as the challenge control (UCC), and uninfected and untreated birds as the negative control (UUC). Drug administration was performed two days prior to infection. 

**Figure 1:**
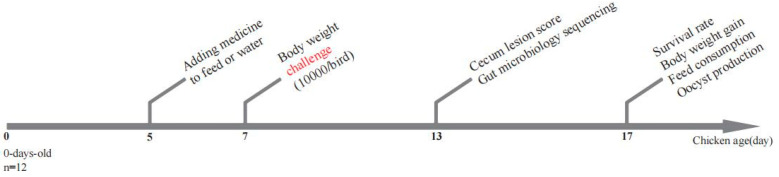
Schematic outline of the experimental design. At 5 days of age, birds were fed with drug-containing feed until the end of the experiments. At 7 days of age, each bird was weighed and infected with 10,000 fresh sporulated *E. tenella* oocysts. At 13 days of age, five birds from each group were executed, and the cecum contents were sampled for microbiota analysis, with the cecum lesions being scored. At 17 days of age, chickens were weighed, and the total number of oocysts was calculated.

After 6 days postinfection (dpi), five birds in each group were killed for cecum sampling and lesion scoring (Figure 1). The cecum lesions were macroscopically evaluated using a scoring system as previously published [36]. Grades from 0 to 4 were given depending on the severity of the lesions. The content of the cecum was rapidly collected in sterile tubes and immediately stored in liquid nitrogen. To calculate the average body weight gain achieved per chicken, chickens were weighed individually on day 7 and day 17 (10 days postinfection, dpi). To assess feed consumption, the amount of feed given to the chickens was weighed daily for each cage. The feed conversion ratio for each cage was calculated as the ratio between the amounts of feed consumed by the chickens per weight gain. Feces from each group were collected for oocyst counting during the 6–10 dpi period (Figure 1). Oocyst counts were repeated three times using the McMaster’s method. Mortality was recorded throughout the experimental period as it occurred, and the exact cause of death was investigated by necropsy examination. 

To evaluate the contribution of each indicator against each group of coccidia in an integrated manner, we calculated the anticoccidial index (ACI) with the following formula: ACI = (%S + %rBGW) − (LI + OI), where %S is the percentage of survival, %rBGW is the percentage of relative weight gain, LI is the lesion index, and OI is the oocyst index. The anticoccidial effect of the drugs was considered excellent when ACI ≥ 180, good when 160 ≤ ACI < 180, fair when 120 ≤ ACI < 160, and no anticoccidial effect when ACI < 120. 

### 2.6. Cecum Microbiota Amplification and Sequencing 

The cecum contents from relatively effective compounds (with >50% reduction of the total oocyst output), UUC, and UCC were subjected for microflora investigation. Total genomic DNA was extracted from the cecum contents of each sample using the E.Z.N.A. ^®^Stool DNA Kit (D4015, Omega, Inc., Norwalk, CT, USA) according to the manufacturer’s instructions. The V3-V4 region of the bacterial 16S rRNA gene was amplified using forward primer 338F (5′-ACTCCTACGGGAGGCAGCA-3′) and reverse primer 806R (5′-GGACTACHVGGGTWTCTAAT-3′). PCR amplicons were purified with Agencourt AMPure Beads (Beckman Coulter, Indianapolis, IN, USA) and quantified using the PicoGreen dsDNA Assay Kit (Invitrogen, Carlsbad, CA, USA). After the individual quantification steps, amplicons were pooled in equal amounts and paired-end 2 × 300 bp sequencing was performed using the Illumina MiSeq platform with MiSeq Reagent Kit v3.

### 2.7. Bioinformatics

The Quantitative Insights Into Microbial Ecology (QIIME, v1.8.0) pipeline was employed to process the sequencing data, as described previously [37]. Briefly, the raw reads obtained from Illumina sequencing were subjected to quality filtering, double-ended sequence splicing, and chimera removal to obtain the final effective clean reads. The remaining clean reads were clustered into operational taxonomic units (OTUs) at 97% sequence identity with UCLUST [38]. A representative sequence was selected from each OTU using default parameters. OTU taxonomic classification was conducted with BLAST using the best hit [39] search against a representative set of sequences from the Greengenes database [40]. OTUs containing less than 0.001% of the total sequences in all samples were discarded. Species richness and species diversity of individual samples were reflected by calculating alpha diversity indices (Chao1 richness estimator, Shannon diversity index, and Simpson index). LEfSe (linear discriminant analysis effect size) was performed to detect differentially abundant taxa among groups using default parameters [41]. All raw sequences were deposited in the NCBI Sequence Read Archive with accession number PRJNA972896. 

### 2.8. Statistical Analysis

All results are expressed as the mean ± standard deviation. Student’s *t*-test within Graphpad Prism 9 was used for statistical analysis for body weight gain, feed conversion rate, lesion score, and oocyst output. Chao1, Shannon, Simpson, and observed species indices were calculated with QIIME, and their comparation among the different groups was conducted via a one-way analysis of variance (ANOVA). *p* < 0.05 indicates a significant difference.

## 3. Results

### 3.1. Effect of Natural Compounds and Their Derivatives on the Growth Performance of Chickens Infected with E. tenella

**Figure 2:**
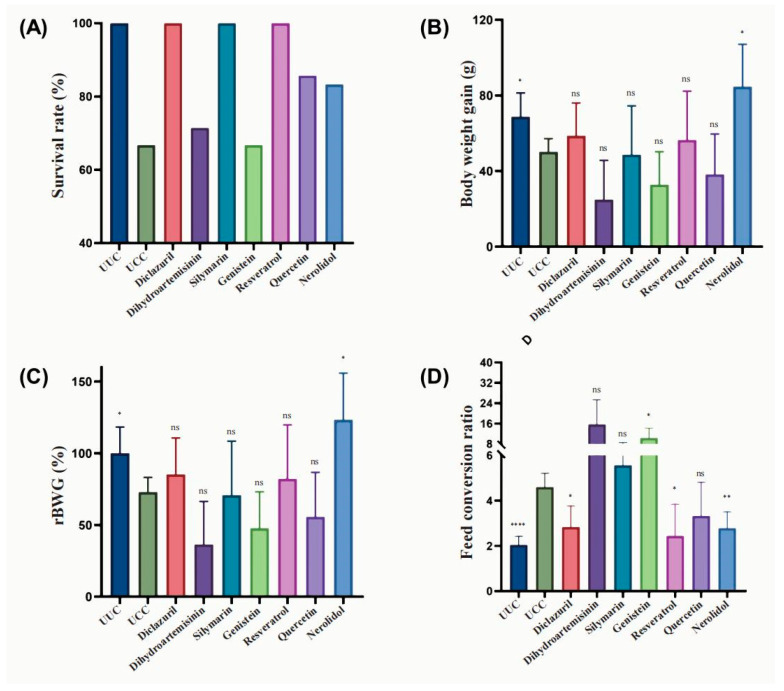
Effect of six natural compounds and their derivatives on the growth performance of chickens infected with *E. tenella*. (**A**) Survival rate of chickens (n = 7) infected with fresh sporulated *E. tenella* oocysts and fed with each of the six natural products, (**B**) body weight gain, (**C**) relative body weight gain, and (**D**) feed conversion ratio. ns: nonsignificant; *: *p* < 0.05; **: *p* < 0.01; ****: *p* < 0.0001.

Challenge with 10,000 *E. tenella* resulted in a UCC mortality rate of ~30%, but this mortality could be completely protected against by 500 ppm of silymarin, 1000 ppm of resveratrol, and 1 ppm of diclazuril and partially protected against by 1000 ppm of quercetin and 500 ppm of nerolidol (Figure 2A). 

For body weight gain, *E. tenella* challenge significantly reduced the average BWG (*p* < 0.05) and could be restored with the anticoccidial diclazuril. Among the six selected natural compounds and derivatives, 500 ppm of nerolidol showed significantly higher BWG and relative BWG (*p* < 0.05) than did the UCC group (Figure 2B,C). Meanwhile, 500 ppm of silymarin and 1000 ppm of resveratrol also showed increased BWG compared to UCC, but they did not show statistical significance (Figure 2B,C). On the other hand, the chickens treated with 75 ppm of dihydroartemisinin had the lowest average body weight gain (*p* > 0.05). 

*E. tenella* infection resulted in a significant elevation in the feed conversion rate, but 1000 ppm of resveratrol (*p* < 0.05) and 500 ppm of nerolidol (*p* < 0.01) additives significantly reversed this effect (Figure 2D). This suggests that these two compounds may have anticoccidial effects or promote growth in chickens. We also noticed a much higher feed conversion rate in the dihydroartemisinin and genistein groups, even well above that of the UCC group (Figure 2D), suggesting that these drugs may have toxic or growth-inhibitory effects on the host at such doses.

### 3.2. Anticoccidial Effects of Natural Compounds and Their Derivatives

**Figure 3:**
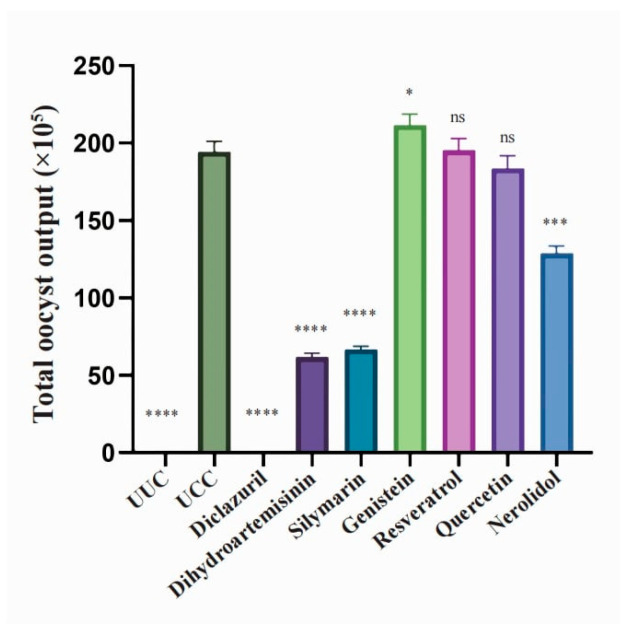
Oocyst output reduction by natural compounds and derivatives. The total amount of feces produced by each group of chickens from 6 to 10 days after the challenge was collected and the total number of oocysts excreted per chicken were calculated. Data were analyzed using Student’s *t*-test. ns: nonsignificant; *: *p* < 0.05; ***: *p* < 0.001; ****: *p* < 0.0001.

No oocysts were found in either the UUC or diclazuril control groups (Figure 3). The oocyst exclusion in the dihydroartemisinin and silymarin groups was only one-third of that in the UCC group (Figure 3), representing the most significant difference (*p* < 0.0001), and that in the nerolidol group was two-thirds of that in the UCC group, representing an extremely significant difference (*p* < 0.001). The oocyst exclusion in the resveratrol and quercetin groups was basically not significantly different from that in the UCC group (Figure 3). 

**Figure 4:**
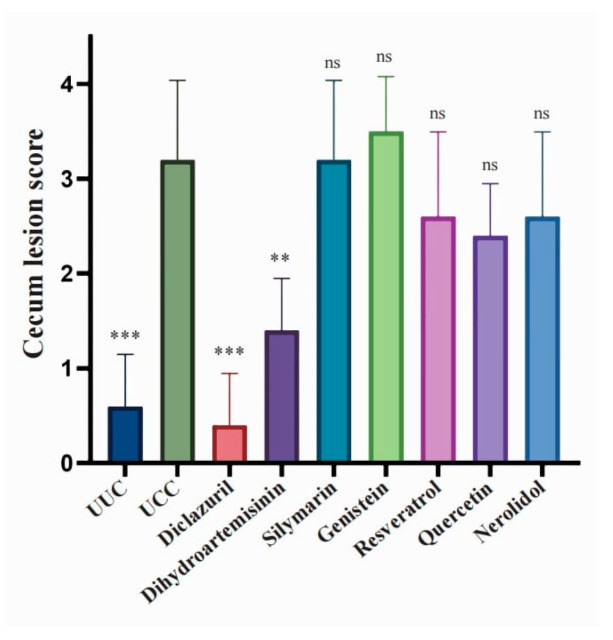
Protection of chicken intestine from *E. tenella* infection by natural compounds and derivatives. On day 6 after *E. tenella* infection, five chickens were killed in each group, and the cecum was removed for pathological observation and scoring (Johnson and Reid, 1970 [36]). Data were analyzed using Student’s *t*-test. ns: nonsignificant; **: *p* < 0.01; ***: *p* < 0.001.

*Eimeria* infects host intestinal epithelial cells and leads to pathological changes in the intestine. Then, six natural compounds and their derivatives were examined for their protective effect on the intestine (Figure S1). Diclazuril treatment completely blocked the cecum lesions caused by *E. tenella* infection, and this protective effect was also significantly observed in the dihydroartemisinin treatment group (*p* < 0.01, Figure 4). However, cecum lesion scores in other treatment groups were not significantly different from those in the UCC group (Figure 4).

**Figure 5:**
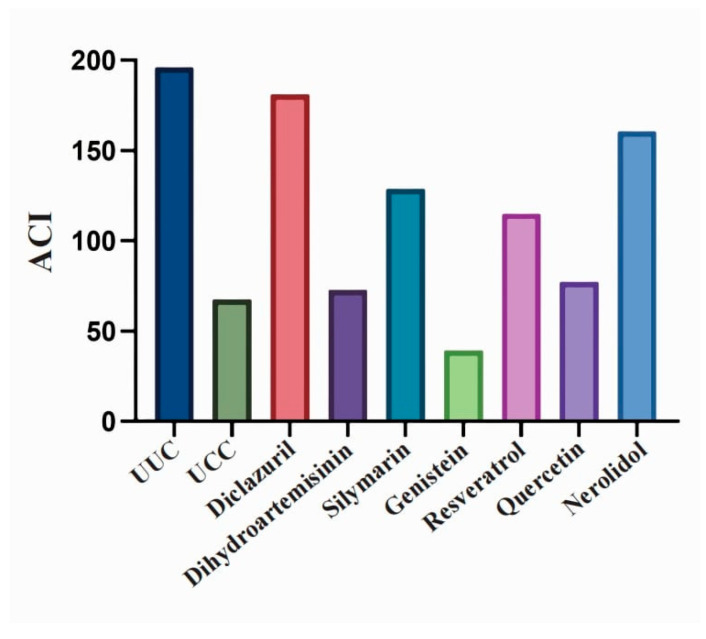
Anticoccidial index of natural compounds and their derivatives. ACI = (%S + %RGW) − (IL + OI), where %S is the percentage of survival, %rBGW is the percentage of relative weight gain (average weight gain of experimental group/average weight gain of UUC), IL is the lesion index (10 × mean of lesion scores of each test group of chickens), and OI is the oocyst index (the number of oocysts in the test group/the number of oocysts in UCC is 0 at 1% or less, 5 at 1–25%, 10 at 26–50%, 20 at 51–75%, and 40 at 76–100%).

The anticoccidial indices of the six natural compounds and derivatives were calculated based on survival, body weight gain, cecum lesion, and oocyst reduction (see detailed data in Table 1). The ACI of UCC was 67.57, while the ACI of the diclazuril control group was greater than 180 (Figure 5), indicating an excellent anticoccidial effect. The ACI of 500 ppm of silymarin and 500 ppm of nerolidol were both greater than 120, while the ACI of the other drugs was lower than 120 (Figure 5), indicating that silymarin and nerolidol have some anticoccidial effect while the other drugs have little anticoccidial effect on coccidia under the judging criteria of the anticoccidial index.

### 3.3. Cecum Microbiota

#### 3.3.1. Alpha Diversity

Based on the analysis of the anticoccidial effects of six natural compounds, we found that 75 ppm of dihydroartemisinin and 500 ppm of silymarin were partially effective in reducing oocyst output (>50% reduction). Thus, we further compared the intestinal microbiota of chickens treated with these two compounds to better elucidate their effects on *E. tenella*. 

**Figure 6:**
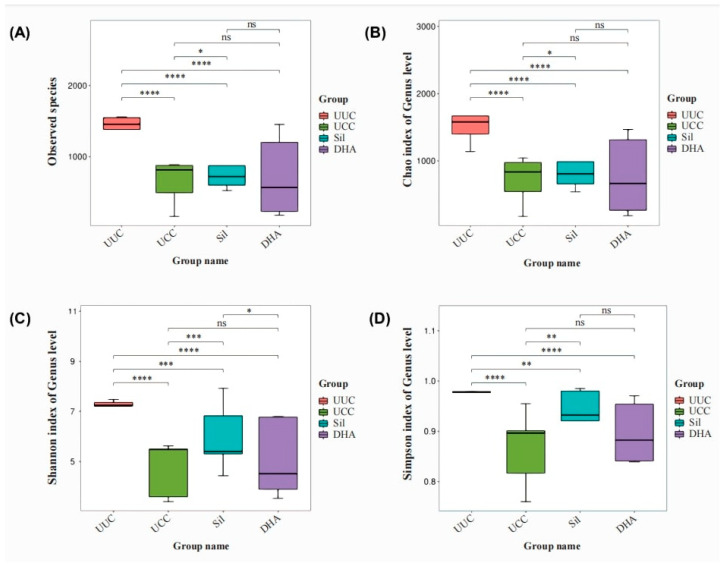
Alpha diversity indexes of the chicken cecum microbiota. Chicken cecum contents were collected 6 days after *E. tenella* infection, and the microflora was determined by 16s DNA sequencing. UUC—untreated and uninfected control; UCC—untreated and challenged control; Sil—chicken infected with *E. tenella* and treated with silymarin; DHA—chicken infected with *E. tenella* and treated with dihydroartemisinin. (**A**) Observed species, (**B**) Chao 1 index, (**C**) Shannon index, and (**D**) Simpson index. * *p* < 0.05, ** *p* < 0.01, *** *p* < 0.001, **** *p* < 0.0001, ns: nonsignificant.

**Figure 7:**
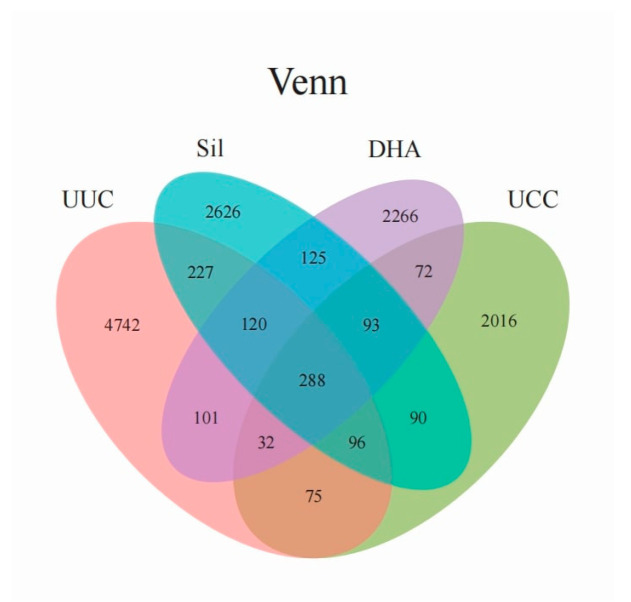
Venn diagram showing the OTUs observed in all groups.

Four indexes were calculated to evaluate the alpha diversity of microflora, including observed species (Figure 6A), Chao 1 (Figure 6B), Shannon index (Figure 6C), and Simpson index (Figure 6D), which provide information regarding microbial richness, biodiversity, and abundant species. The highest indexes of observed species, Chao 1, Shannon. And Simpson were observed in UUC compared to the challenge groups (UCC, Sil. and DHA). The observed species, Chao 1, Shannon. and Simpson indexes were also significantly higher in Sil than in UCC, suggesting that Sil may contribute to the recovery of the cecum microbiota after *E. tenella* infection. However, the indexes of alpha diversity in the comparison of UCC with DHA and Sil with DHA were not significant (Figure 6), suggesting that DHA may have a limited role in altering intestinal microbiota after *E. tenella* infection. We also found 5681, 2762, 3665. And 3097 OTUs in the UUC, UCC, Sil. and DHA groups, respectively, of which 288 were common in all experimental groups. Moreover, a total of 11,650 unique OTUs were detected within UUC, UCC, Sil, and DHA (4742, 2016, 2626, and 2266, respectively; Figure 7).

#### 3.3.2. Effects of Treatments on Bacterial Abundance at the Phylum Level 

**Figure 8:**
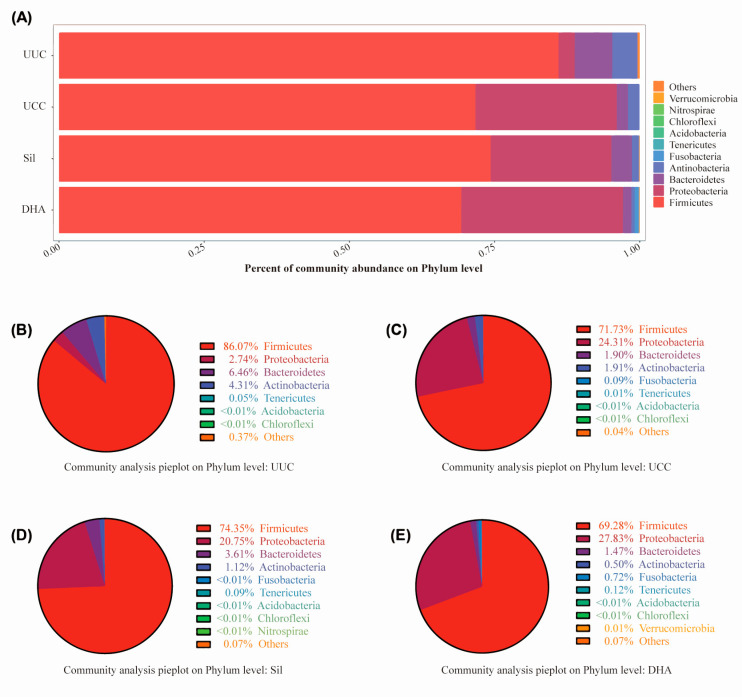
Relative abundance of taxa at the phylum level. (**A**) Relative abundance of affected taxa at the phylum level among all groups. The total affected percentages for each phylum in the UUC (**B**), UCC (**C**), Sil (**D**), and DHA (**E**) groups are shown as pie charts.

To further understand the microbial differences between groups, we next assessed the taxonomic composition of the cecum microbiota (Figure 8). At the phylum level, Proteobacteria significantly increased in the challenge groups (UCC, Sil, and DHA), while bacteria in the phylum of Firmicutes, Bacteroidetes, and Actinobacteria decreased after the challenge. Among all the phyla, Firmicutes comprised the dominant population and was the most severely affected phylum after *E. tenella* infection. Moreover, the distribution of Firmicutes, Bacteroidetes, and Proteobacteria in the silymarin-treated group had a higher similarity to that of UUC compared to UCC and DHA.

#### 3.3.3. Effects of Treatments on Bacterial Abundance at the Genus Level

**Figure 9:**
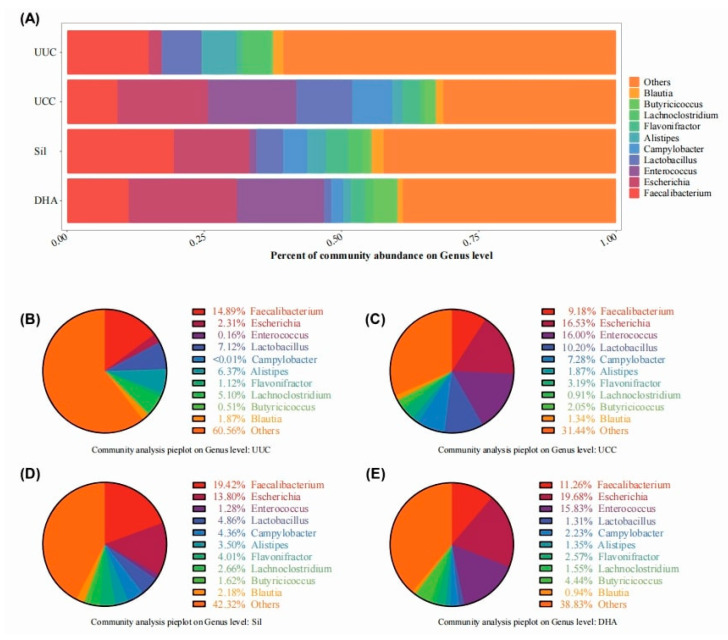
Relative abundance of taxa at the genus level. (**A**) Relative abundance of affected taxa at the genus level among all groups. The total affected percentages of each genus in the UUC (**B**), UCC (**C**), Sil (**D**), and DHA (**E**) groups are shown as pie charts.

We further compared the bacterial composition in feces of all experimental treatments at the genus level (Figure 9). *Escherichia coli* (belonging to the Proteobacteria phylum) increased significantly after infection (Figure 9A). *E. tenella* infection reduced the population of Faecalibacterium, but Sil and DHA treatments recovered its abundance (Figure 9); *E. tenella* infection largely increased the population of Enterococcus (belonging to the Firmicutes phylum), but Sil treatment significantly recovered its abundance, while DHA was unable to (Figure 9). At the level of genera, the microflora composition of the silymarin-treated group also remained more consistent with that of the UUC group than that of the UCC and DHA groups (Figure 9).

**Figure 10:**
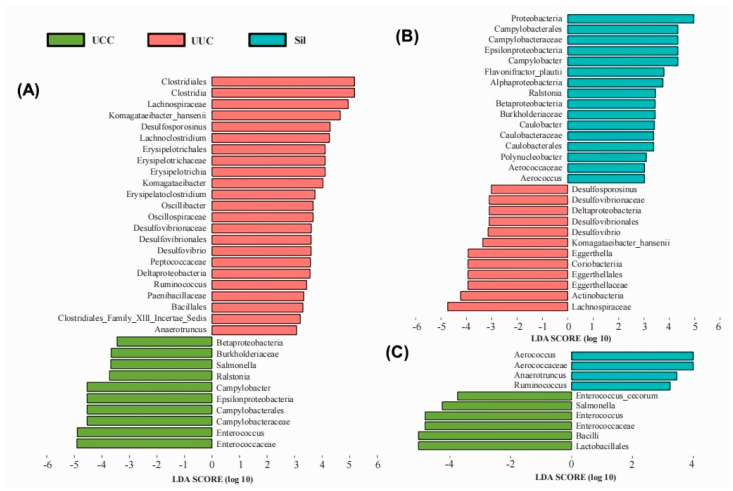
Differences in microbial abundance in different groups. Linear discriminant analysis effect size (LEfSe) was used to analyze the differences in microbial abundance in (**A**) UCC versus UUC, (**B**) UUC versus Sil, and (**C**) UCC versus Sil (n = 5 samples/group) (LDA = 3).

LEfSe analyses were also performed to fully understand the influence of natural compounds and their derivatives on the gut microbiota during Eimeria infections. The results of the LEfSe analysis showed that several genera were affected by the addition of natural compounds and derivatives as well as by inoculation with Eimeria infections (Figure 10). Compared to UUC, UCC showed a significant decrease in the abundance of *Clostridiales*, *Clostridia*, *Lachnospiraceae*, *Komagataeibacter_hansenii*, *Desulfosporosinus*, *Lachnoclostridium*, *Erysipelotrichales*, *Erysipelotrichaceae*, *Erysipelotrichia*, *Komagataeibacter*, *Erysipelatoclostridium*, *Oscillibacter*, *Oscillospiraceae*, *Desulfovibrionaceae*, *Desulfovibrionales*, *Desulfovibrio*, *Peptococcaceae*, *Deltaproteobacteria*, *Ruminococcus*, *Paenibacillaceae*, *Bacillales*, *Clostridiales_Family_Xlll_Incertae_Sedis*, and *Anaerotruncus*, while increased abundance of *Betaproteobacteria*, *Burkholderiaceae*, *Salmonella*, *Ralstonia*, *Campylobacter*, *Epsilonproteobacteria*, *Campylobacterales*, *Campylobacteraceae*, *Enterococcus*, and *Enterococcaceae* was detected (Figure 10A). Compared to UUC, Sil significantly decreased the abundance of *Desulfosporosinus*, *Desulfovibrionaceae*, *Deltaproteobacteria*, *Desulfovibrionales*, *Desulfovibrio*, *Komagataeibacter_hansenii*, *Eggerthella*, *Coriobacteriia*, *Eggerthellales*, *Eggerthellaceae*, *Actinobacteria*, and *Lachnospiraceae*, while increasing the abundance of *Proteobacteria*, *Campylobacterales*, *Campylobacteraceae*, *Epsilonproteobacteria*, *Campylobacter*, *Flavonifractor_plautii*, *Alphaproteobacteria*, *Ralstonia*, *Betaproteobacteria*, *Burkholderiaceae*, *Caulobacter*, *Caulobacteraceae*, *Caulobacterales*, *Polynucleobacter*, *Aerococcaceae*, and *Aerococcus* (Figure 10B). In addition, *Aerococcus*, *Aerococcaceae*, *Anaerotruncus,* and *Ruminococcus* were more enriched in the infected chickens supplemented with silymarin in the feed compared to UCC (Figure 10C). In this experiment, we also performed the LEfSe analysis in the comparison between UCC and DHA, but there were no significantly different species that could satisfy the condition of LDA = 3.

## 4. Discussion

In this experiment, the anticoccidial effects of six natural products and their derivatives were evaluated in terms of survival rate, body weight gain, feed conversion rate, total oocyst production, and cecum lesions. In addition, the influence of silymarin and dihydroartemisinin on the cecum microbiota of chickens infected with *E. tenella* was determined.

Silymarin is a natural active substance obtained from the dried fruits of silymarin, a plant of the Asteraceae family, with good antitumor, antioxidant, and gastrointestinal tract protective effects [42]. In this experiment, we found that 500 ppm of silymarin ensured the survival of chickens and significantly reduced the oocyst output by two-thirds. At the doses used, it did not negatively affect body the weight gain and feed conversion ratio in chickens. This suggests that silymarin can be used in combination with some natural products [10], thus allowing for the control of chicken coccidiosis while still ensuring the performance of chickens. In addition, the results of this study show that silymarin has a better resistance effect on the disturbance of intestinal microorganisms by coccidia in chickens and can maintain the stability of intestinal microorganisms to a certain extent.

Dihydroartemisinin is a derivative of artemisinin with higher antimalarial activity than artemisinin [43]. In the present experiment, 75 ppm of dihydroartemisinin played a moderate role in reducing the total number of oocysts output as well as the cecum lesion score, which is consistent with the anticoccidial effect of artemisinin [2]. However, the body weight gain and feed conversion ratio of dihydroartemisinin were not significantly different from those of UCC. Dihydroartemisinin treatment resulted in an increased feed conversion ratio, even much higher than that of UCC. This suggests that the current dose may have toxic effects on chickens and reduce their growth performance [44]. Given the potential toxicity issues associated with dihydroartemisinin in chickens, a full toxicity evaluation, including histopathological examinations and biochemical analyses, is essential to assess its safety profile in chickens in further study. 

Nerolidol is a naturally occurring sesquiterpene alcohol present in the essential oils of various plants with a floral odor [45,46], which has a good potential in antimalarial treatment. In this experiment, 500 ppm of nerolidol showed good improvements in terms of body weight gain and feed conversion ratio, and it was the only drug in this experiment that led to significantly higher body weight gain than did UUC. Nerolidol also reduced the oocyst output in infected chickens. In terms of cecum lesions, nerolidol scored lower than did UCC, but the difference was not significant, which could be due to underdosing. Overall, nerolidol showed good results in the ACI evaluation, achieving moderate effectiveness as an anticoccidial agent. Follow-up studies may explore the appropriate dose of nerolidol for administration as well as its combination with other drugs to achieve better anticoccidial effects in chickens.

Quercetin, genistein, and resveratrol were selected to investigate their anticoccidial activity based on their effects on other apicomplexan protozoan parasites such as *T. gondii* or *P. falciparum* [25,26,27,28]. In comparison with those used in related studies, the drugs used in this study took quite high drug doses but did not show significant anticoccidial effects. We suspect that their mechanism of action against *Toxoplasma* and *Plasmodium* is different from that of *E. tenella* and/or that these compounds have different metabolizing pathways in chickens. Subsequent studies on the anticoccidial effects of these drugs should be preceded by an exploration of their appropriate concentrations of action.

The abundance and diversity of microorganisms in the gastrointestinal tract are closely related to the health of chickens. Clostridial overgrowth with subsequent deep ulceration of the intestinal mucosa is a major concern in coccidiosis [47], and other bacteria in this phylum, such as Enterococcus [48] and Faecalibacterium, also aggravate/alleviate intestinal burdens [49]. A significant reduction in flora diversity [20] and a significant reduction in Firmicutes populations have been reported in the cecum mucosa of *E. tenella* infected chickens [50,51], with a consequent reduction in feed conversion [52]. Our data well support these findings and indicate that silymarin can partly recover the microflora from the coccidiosis. For example, *E. tenella* infection reduced the population of Faecalibacterium and largely increased the population of Enterococcus, but silymarin treatment significantly recovered the abundance of the healthy control (Figure 9). Faecalibacterium, such as *F. prausnitzii*, plays a crucial role in maintaining gut physiology and host well-being. It is one of the main butyrate producers found in the intestine. It is a major source of energy for the colonocytes and has protective properties against colorectal cancer and inflammatory bowel diseases. Faecalibacterium supernatant has also been shown to attenuate the severity of inflammation by releasing metabolites that enhance intestinal barrier function and affect paracellular permeability [53]. Enterococcus produces a protease that could contribute to the development of experimental colitis by impairing epithelial barrier function [54]. Enterococcus also exhibits an augmented ability to degrade collagen and activate intestinal tissue matrix metalloproteinase 9 (MMP9), which contributes to the development of anastomotic leakage [55]. The use of silymarin has a positive effect on the intestinal tract via both the mechanisms of microbial action on the intestinal tract and the maintenance of intestinal microbial stability.

## 5. Conclusions

This study focused on the anticoccidial effects of six natural compounds and derivates. Our results showed that silymarin, dihydroartemisinin, and nerolidol have a partial effect against *E. tenella* under certain concentrations and that both silymarin and dihydroartemisinin could partially restore the microbiota after E. tenella challenge. This study provides basic knowledge about the anticoccidial effects of such natural com-pounds/derivates.

## Figures and Tables

**Table 1:** Summary of indicators for assessing coccidia in chickens.

	UUC	UCC	Diclazuril	Dihydroartemisinin	Silymarin	Genistein	Resveratrol	Quercetin	Nerolidol
Survival rate	100.0	71.42	100.0	71.42	100.0	66.67	100.0	85.71	83.33
BWG	68.73 ± 12.59 *	50.15 ± 6.99	58.60 ± 17.41	24.97 ± 20.71	48.62 ± 25.93	32.78 ± 17.49	56.43 ± 25.88	38.22 ± 21.39	84.71 ± 22.37 *
rBWG	100.00	73.00 ± 10.18	85.30 ± 25.35	36.34 ± 30.14	70.77 ± 37.74	47.71 ± 25.46	82.14 ± 37.68	55.63 ± 31.14	123.31 ± 32.56 *
FCR	2.03 ± 0.38 ****	4.59 ± 0.62	2.83 ± 0.92 *	15.63 ± 9.72	5.55 ± 3.06	10.30 ± 3.95 *	2.44 ± 1.40 *	3.32 ± 1.49	2.78 ± 0.72 **
Total oocyst output (*10^5^)	0 ****	194.24 ± 6.93	0 ****	61.73 ± 2.55 ****	66.86 ± 1.82 ****	211.58 ± 7.07 *	195.35 ± 7.50	183.49 ± 8.38	128.69 ± 4.86 ***
Cecum lesion score	0.60 ± 0.54 ***	3.20 ± 0.84	0.40 ± 0.54 ***	1.40 ± 0.54 **	3.20 ± 0.83	3.50 ± 0.57	2.60 ± 0.89	2.40 ± 0.55	2.60 ± 0.89
ACI	196.0	67.57	181.3	72.76	128.7	39.2	115.0	77.31	160.6

Notes: * *p* < 0.05, ** *p* < 0.01, *** *p* < 0.001, **** *p* < 0.0001.

## Data Availability

Data are contained within the article and Supplementary Materials. All raw sequences were deposited in the NCBI Sequence Read Archive with accession number PRJNA972896.

## Supplementary Materials

The following supporting information can be downloaded at https://www.mdpi.com/article/10.3390/microorganisms12030601/s1, Figure S1: Photos of the intestinal lesions.
