# Supplementary figures and images for: Effects of Six Natural Compounds and Their Derivatives on the Control of Coccidiosis in Chickens
Source: Microorganisms. 2024 Mar 17;12(3):601. doi: 10.3390/microorganisms12030601 (PMC10975649; doi:10.3390/microorganisms12030601)

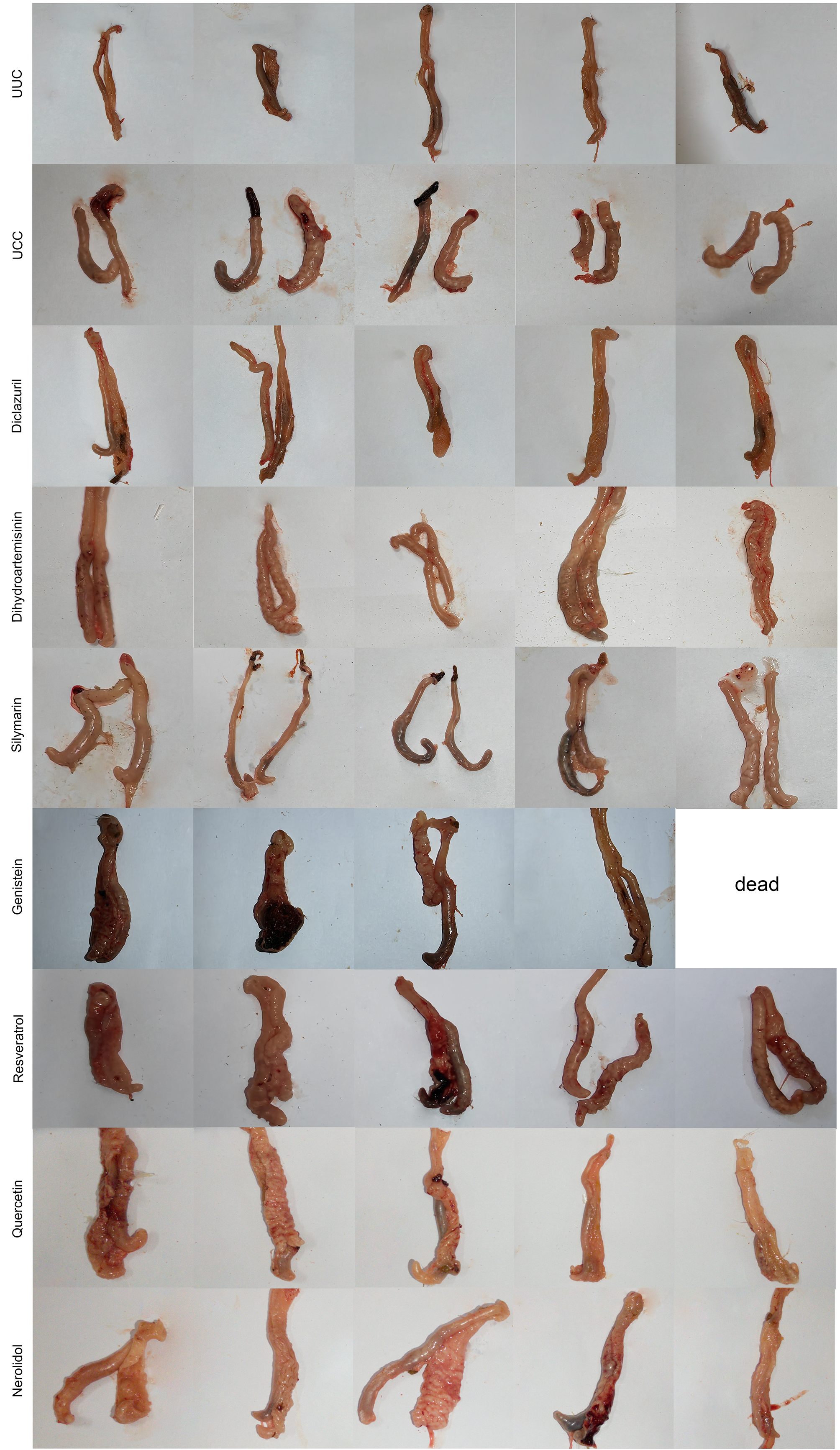

Supplement: Supplementary file 1 [file microorganisms-12-00601-s001.zip › microorganisms-2904399-supplementary.tif]
